# Generation of Prior Information in a Dual-Mode Microwave-Ultrasound Breast Imaging System

**DOI:** 10.3390/s22187087

**Published:** 2022-09-19

**Authors:** Hannah Fogel, Max Hughson, Mohammad Asefi, Ian Jeffrey, Joe LoVetri

**Affiliations:** 1 Department of Electrical and Computer Engineering, University of Manitoba, Winnipeg, MB R3T 5V6, Canada; 2 Antec Controls, Winnipeg, MB R2K 3Z9, Canada; 3 AGCO Winnipeg, Winnipeg, MB R3T 2T8, Canada

**Keywords:** microwave imaging, ultrasound imaging, breast imaging, breast cancer, medical imaging, multi-modal

## Abstract

A new breast imaging system capable of obtaining ultrasound and microwave scattered-field measurements with minimal or no movement of the breast between measurements has recently been reported. In this work, we describe the methodology that has been developed to generate prior information about the internal structures of the breast based on ultrasound data measured with the dual-mode system. This prior information, estimating both the geometry and complex-valued permittivity of tissues within the breast, is incorporated into the microwave inversion algorithm as a means of enhancing image quality. Several techniques to map reconstructed ultrasound speed to complex-valued relative permittivity are investigated. Quantitative images of two simplified dual-mode breast phantoms obtained using experimental data and the various forms of prior information are presented. Though preliminary, the results presented herein provide an understanding of the impacts of different forms of prior information on dual-mode reconstructions of the breast and can be used to inform future work on the subject.

## 1. Introduction

The use of microwaves to quantitatively image the complex-valued permittivity of the breast ($\hat{\epsilon }=\epsilon^{\prime }-j\epsilon^{\prime \prime }$) has been studied for several decades. It is well-documented in the literature that the complex-valued permittivity of cancerous tissue is notably higher than that of healthy breast tissue. Thus, as compared to some other modalities, microwave imaging (MWI) provides good specificity but lacks the resolution required to detect early-stage breast cancer [1,2,3]. The ability to produce quantitative images of the physical properties of the breast is a strong benefit of MWI compared to traditional X-ray mammography or ultrasound imaging, and so research into techniques that can improve the quality of MW images is ongoing [4].

One possible method for enhancing the resolution of microwave images is to incorporate prior information about the object of interest (OI) into the inversion algorithm. Several past studies have investigated methods of extracting prior information from other imaging modalities [5,6,7,8], including microwave radar (MWR), magnetic resonance imaging (MRI), and ultrasound, as well as integrating this information into microwave inversion algorithms [9,10,11,12]. The use of high-resolution geometrical prior obtained from MRI or USI has been shown to allow for smaller tumours to be identified, and with better accuracy than using MWI alone [8,9,10]. The imaging modality chosen to create the prior information can impact both the type and quality of the prior information, as well as the practical design of a dual-mode imaging system. A significant drawback of MWR is its inability to produce prior information with high spatial resolution, due to the long wavelengths used. MRI offers high resolution; however, from a system design perspective, introduces significant practical challenges, as well as the need for expensive equipment [8]. Qualitative ultrasound imaging (USI), commonly used in a clinical setting, is often used to complement traditional X-ray mammography [13,14,15]. This technique, which uses a hand-held transducer wand to scan the breast, requires a trained operator whose skill level may affect the quality of the resulting images [13,14]. Conversely, *quantitative* USI systems typically perform automated scans of the whole breast and help distinguish benign, fluid-filled cysts from solid masses, which show up similarly in X-ray mammography but have different acoustic speeds [13]. Another benefit of USI is that compared to MWR, the shorter wavelengths allow for higher spatial resolution prior information to be obtained.

With these factors considered, the modality chosen to complement MWI in this work is a novel, inexpensive quantitative USI system [16]. Previous works published by our group and others on incorporating US-derived prior information into MW inversion algorithms have relied on numerical data [11,17,18,19]. An exception is [9], in which a 2D dual-mode MW-US system was used to produce cross-sectional images of the breast. The experimental 3D dual-mode MW-US imaging system referenced in the current work is the first of its kind, to the best of the authors’ knowledge [16].

To validate the new system, two dual-mode breast phantoms (having acoustic and dielectric properties similar to those of real breast tissues) were developed. A quantitative 3D ray-based US inversion algorithm was used to produce images of the acoustic speed of the phantoms [20]. The prototype USI system has limited resolution due to the small number of piezoelectric transducers used but can provide an estimate of the shape, size, and acoustic speed of tissue regions within the breast. This information is incorporated into our existing finite element method contrast source inversion (FEM-CSI) algorithm to help overcome the non-linearity and ill-posedness of the problem. The MWI system used as part of the new dual-mode system has been previously reported in [21,22].

A system-level description of the new dual-mode system is provided in Section 2; the individual USI and MWI systems are described in Section 2.1 and Section 2.2, respectively. Properties of the dual-mode tissue-mimicking breast phantom used in this work are given in Section 2.3. Section 2.4 provides an overview of the USI and MWI inversion algorithms, with details of the methodology for incorporating US-derived prior information into FEM-CSI provided in Section 2.5. USI, MWI, and dual-mode inversion results are presented in Section 3. Finally, Section 4 contains a discussion of the results and outlines our group’s plans for future work.

## 2. Materials and Methods

**Figure 1:**
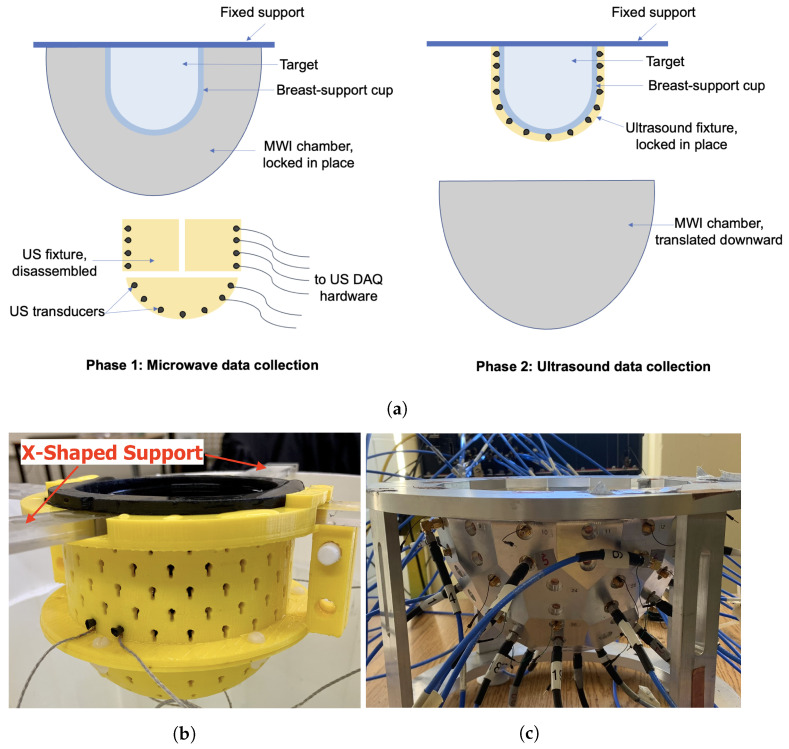
Dual-mode data collection. (**a**) Conceptual diagram. (**b**) US fixture positioned with breast phantom and X-shaped support. (**c**) MWI chamber.

As reported in [16,23], the integrated MWI-USI system makes use of an US fixture used in tandem with a MWI chamber. The goal of an integrated dual-mode imaging system is to acquire data from two different imaging modalities without having to move the OI from one system to another. Ideally, the OI should not have to be repositioned at all between scans. The schematic in Figure 1a outlines the method by which data can be collected without a registration error. The “breast-support cup” referred to in the figure is a flexible, cup-like mould that surrounds the breast being imaged with minimal compression. For the results shown in this paper, the skin layer of the breast phantom also serves as the breast-support cup. The following are the design features of the breast-support cup. It should:

1. Hold the breast in a known, fixed position and geometry while imposing minimal compression on the breast;
2. Allow the US transducers to make direct contact with its surface, via slight compression or the use of an ultrasonic coupling gel;
3. Have favourable acoustic properties: limited attenuation and limited reflection at the skin interface so that sufficient acoustic energy penetrates the breast; and
4. Have favourable MW properties that allow MW energy to interrogate the breast while being easily incorporated in the MWI inversion model (as prior information).

In the final design, mechanical infrastructure will allow the lowering of the MWI chamber so that the USI fixture may be fastened to the breast-support cup. For results shown in this work, data for both modalities were collected with the target held by an X-shaped plexiglass support. The US fixture locks into the X-shaped support and markings were made on the top of the MWI chamber so that the support could be placed consistently at the same location and the OI’s position could be known exactly. After MWI data were acquired, the OI and support were carefully transported to a temporary container where the US fixture was attached.

**Figure 2:**
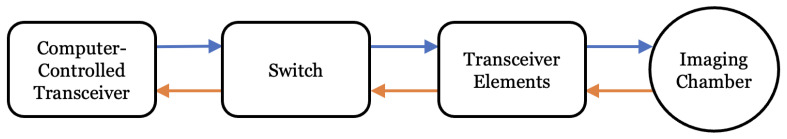
Flowchart of MWI and USI system components. For USI, the computer-controlled transceiver (CCT) consists of a PC, AWG, and digital oscilloscope; the transceiver elements are US transducers. For MWI, the CCT is a VNA and the transceiver elements are magnetic field probes.

The flow of data through each of the USI and MWI systems is detailed in Figure 2. For USI, the computer-controlled transceiver (CCT) consists of a personal computer (PC) connected to a digital oscilloscope and arbitrary waveform generator, as described further in Section 2.1. For MWI, the CCT is a vector network analyzer (VNA), as described in the upcoming Section 2.2. The switch routes the excitatory signal from the CCT to the transmitting transceiver element: a piezoelectric transducer in the case of USI and a magnetic field probe in the case of MWI. For both modalities, the transceiver elements radiate one by one, and scattered field data are collected by all other transceivers. The switch directs measured data back to the CCT, where it is stored for later use in image reconstruction.

### 2.1. Ultrasound System Description

The US data acquisition system consists of the following parts:

- An arbitrary waveform generator (AWG), comprised of four PC-based waveform generator cards (Signatec PXDAC4800), each with four channels. A pulsed sinusoid truncated after five periods was used to drive the US transducers at their observed resonant frequency of 1.4 MHz.
- A 32-channel digital oscilloscope, composed of four PC-based oscilloscope cards (GaGe Octopus 8387 CompuScope digitizer boards), each with 8 channels.
- A custom switch module that routes the transmitted signal from the waveform generator to the specified transducer and received signals from transducers to the corresponding oscilloscope channels.
- Custom MATLAB control software that coordinates the above components, including a graphical user interface (GUI).
- A novel, 3D-printed fixture that holds the 64 piezoelectric transducers in direct contact with the breast-support cup.

The US transducers (Sonometrics Corporation, London, Canada) are single-element piezoelectric transducers. Each element consists of a cylindrical lead zirconate titanate (PZT-5H) crystal, connected to a twisted pair of wires. The crystals are dipped in epoxy to provide better coupling to the background material, assumed to be water or similar. The twisted pair is surrounded by a ground shield and encased inside a waterproof coating. The usable bandwidth of the transducers is approximately 1.0–1.8 MHz.

The US fixture is made up of two hollow semi-cylindrical pieces plus a semi-spherical cup, housing 64 transducers arranged in a helical pattern. Small, keyhole-shaped cutouts hold the transducers against the breast-support cup. The three-piece fixture is fastened together using plastic pin connectors and nylon screws and is easily taken apart to remove the fixture from the target after data collection. The fixture is designed to fit snugly around a flexible capped-cylindrical container that doubles as the breast-support cup and the skin layer of the breast phantom [24]. US coupling gel is applied to the outer surface of the breast-support cup to help facilitate the transmission of US energy into the phantom. An image of the fixture is shown in Figure 1b.

### 2.2. Microwave System Description

The microwave measurement system used here is the same air-based, flat-faceted, quasi-resonant metallic chamber published in [21]. This system has several advantages, including being air-based as well as the flat facets being easy to model in the FEM-CSI inversion model [22]. An air-based system simplifies registration of the OI between the two imaging systems, as the variable buoyancy of breasts can lead to the unpredictability of the exact position and shape of the breast in a fluid-filled chamber [21]. S-parameter measurements are made using a VNA connected to magnetic field probes installed on 24 of the facets of the metallic chamber. MW measurements are taken while the breast is positioned within the breast-support cup but with the US fixture removed. A photograph of the chamber is shown in Figure 1c.

### 2.3. Breast Phantoms

**Figure 3:**
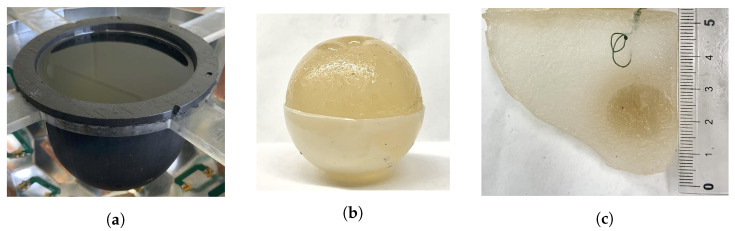
Dual-mode breast phantom components. (**a**) Skin and fat. (**b**) Spherical tumour in the bottom half of the mould. (**c**) Cross-section of fibroglandular with embedded ellipsoidal tumour. Ruler in cm.

The first dual-mode phantom (Phantom 1) was a simplified breast model consisting of skin, fat, and tumour regions only [16]. The skin-mimicking material was a 10 cm diameter flexible capped-cylindrical shell [24]. Fat was modelled using canola oil. A 3.5 cm diameter spherical inclusion was added to the fat region to mimic a tumour. The second, more realistic breast phantom (Phantom 2) used the same skin- and fat-mimicking materials as Phantom 1, but also included an asymmetric fibroglandular region with an embedded tumour [16,23]. For both breast phantoms, the fibroglandular and tumour regions were composed of gelatin-based materials. The recipes were adapted from the acoustic phantom recipe published in [25] to also exhibit dielectric properties in the same ranges as real breast tissues. The exact ingredients and the physical properties at the imaging frequencies for each phantom tissue are given in Table 1. Photographs of the phantom components are provided in Figure 3. For the gelatin-based materials (fibroglandular and tumour phantoms), the listed ingredients were mixed together and then heated in a microwave oven or on a hot plate until just before boiling. For Phantom 2, the 1-propanol was added slowly to the heated solution while stirring constantly to ensure that the mixture was completely dissolved [16]. The heated solutions were left to solidify at room temperature for several hours in custom-made moulds of the desired shapes. The phantom components were removed from the moulds prior to data collection. To embed the tumour inside the fibroglandular region of Phantom 2, the tumour was formed first and hung by a thread within the fibroglandular mould. The liquid fibroglandular-mimicking material was poured over the top of the solidified tumour and left to set. Two loops of thread were placed protruding from the top of the setting fibroglandular material, allowing it to be suspended in the liquid fat region for imaging. One of these threads is visible near the top of Figure 3c. A similar technique was used to suspend the tumour in the fat for Phantom 1.

The physical properties of the breast phantoms used are approximations of the parameters that might be expected for actual tissue. Published values of acoustic speed at 1.5 MHz and relative permittivities at 1.1 GHz of real breast tissues are provided for reference in italics in Table 1 [13,26]. It should be noted that the measured sound speed values for the fibroglandular and tumour tissues are nearly the same, considering the accuracy of the US system. This puts the USI algorithm at a disadvantage for detecting the tumour in Phantom 2. However, these properties are still suitable for the present study, in which we investigate whether low spatial resolution US reconstructions may be used to enhance MWI. The discrepancy in measured permittivity values for the tumour-mimicking material in the two phantoms may be due to slight variations in manufacturing in combination with measurement error. Dielectric property measurements were made using an Agilent 85070E slim form dielectric probe (Agilent Technologies, Santa Clara, CA, USA) that has a reported accuracy of ±5%. Measurements of acoustic speed were made using the experimental system and the through-transmission technique. Measured permittivity values as functions of frequency between 0.9 and 2 GHz for the tissue-mimicking materials are available in [16,23].

### 2.4. US and MW Inversion Algorithms

The dual-mode setup described at the start of Section 2 could be utilized to acquire several forms of US and MW data. For the imaging results presented herein, the US data consists of time-domain pulsed transmission waveforms while the MW data consists of single frequency phasor values for each transmitter–receiver pair. From the time-domain US waveforms, time-of-flight (TOF) between transmitter and receiver pairs is extracted using one of several well-known algorithms, such as MER [27,28] or AIC [29]. The TOF data are then processed to build a 3D speed-of-sound image using a ray-based, whole-domain basis function algorithm. Using this method, the sound speeds within the phantom are represented as a weighted sum of the whole-domain basis functions, and the algorithm solves for the weights. The basis functions used are polynomials, and their order can be varied. Full details of the sound speed inversion algorithm are available in [20].

For the MW inversions, we utilized our FEM-CSI algorithm with prior information acquired from the US speed image [30,31]. Several methods of using prior information in the FEM-CSI algorithm have been studied in the past [11,32,33]. In this work, we simply utilize an approximation of the complex-valued permittivity obtained from the acoustic-speed reconstruction as an initial guess. Scattered field data were created by subtracting incident field measurement data $U^{inc}$ for some inhomogeneous background from the measured total field data with the OI present $U_{OI}^{tot}$. Calibration was performed on the scattered field data using calibration coefficients obtained using a skin-fat phantom (the same, or similar, breast phantom without the tumour) [22,34]. The calibrated scattered field data *d* that were inverted by CSI were obtained as,

(1) $$d≜H^{sct}=\frac{H_{cal\_obj}^{tot}}{U_{cal\_obj}^{tot}}*\left(U_{OI}^{tot}-U^{inc}\right)$$

where *U* denotes measured fields and *H* denotes synthetic fields modelled with the FEM forward solver [34]. $U_{cal\_obj}^{tot}$ is the synthetic data generated by modelling the calibration object using FEM. The fractional term, $H_{cal\_obj}^{tot}/U_{cal\_obj}^{tot}$ constitutes the calibration coefficients, *C*. For the results shown in this paper, the calibration object and incident field background are the same, i.e., $U_{cal\_obj}^{tot}=U^{inc}$. In this case, Equation (1) is equivalent to just calibrating the total field and subtracting a numerical incident field [34]:

(2) $$d=CU^{tot}-H^{inc}.$$

### 2.5. Converting Sound Speed to Permittivity

One of the main contributions of this work was in finding a methodology for incorporating the US-derived prior information into the MWI algorithm. In this section, we describe our initial study of possible methods for generating prior information from the US speed images. In Section 3, we show the impact that each of these methods has on the final inversion results.

To use information from an US image in FEM-CSI, the reconstructed sound speeds must be converted to complex-valued permittivities. Three different functions for mapping sound speeds to permittivities were investigated:

1. **Segmented mapping**—a k-means clustering algorithm was used to segment the sound speeds into two or more regions, e.g., fat and fibroglandular. Representative complex permittivity values for the tissues are assigned to the respective regions.
2. **Linear mapping**—reconstructed sound speed values *c* are linearly re-scaled to ranges representative of real and imaginary parts of complex permittivity, using the following equations:
  (3) $$\epsilon^{\prime }=\epsilon_{min}^{\prime }+\frac{c-min(c)}{max(c)-min(c)}\left(\epsilon_{max}^{\prime }-\epsilon_{min}^{\prime }\right)$$
  (4) $$\epsilon^{\prime \prime }=\epsilon_{min}^{\prime \prime }+\frac{c-min(c)}{max(c)-min(c)}\left(\epsilon_{max}^{\prime \prime }-\epsilon_{min}^{\prime \prime }\right)$$
3. **Tissue-range mapping**—a range of values of each physical property for each tissue type is set, based on the representative complex permittivity values. For each reconstructed value of speed, the distance from the mean of the corresponding tissue-dependent range is calculated. Complex permittivity is then assigned as the same relative distance from the mean for the given tissue type. This type of mapping is based on the tissue-dependent mapping technique described in [26].

For the results shown herein, the representative complex permittivity values for all mappings are obtained from the measured properties of the phantoms. The tissue-dependent ranges used for Phantom 1 are provided in Table 2. The ranges used for Phantom 2 differ slightly and are available in [16].

## 3. Results

### 3.1. Ray-Based Ultrasound Speed Reconstruction

**Figure 4:**
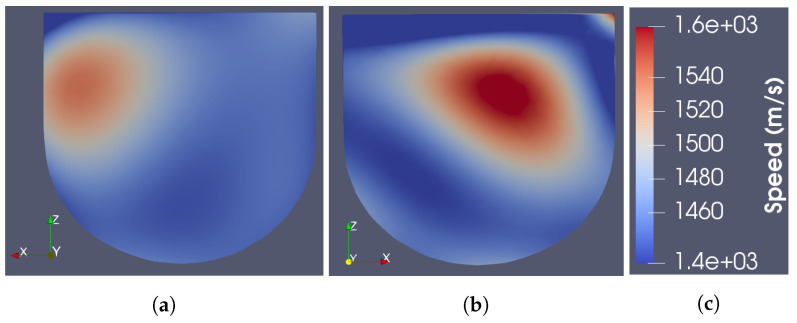
Cross-section of US-only images. (**a**) Phantom 1. (**b**) Phantom 2. (**c**) Colour bar.

**Figure 5:**
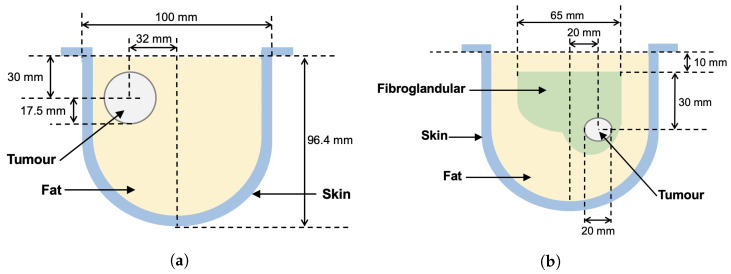
Breast phantom schematic diagrams. Measured positions of tissue regions are approximate. (**a**) Phantom 1. (**b**) Phantom 2.

As described in more detail in [20], the ray-based sound speed imaging algorithm uses whole-domain polynomial basis functions to reconstruct the sound speed inside the imaging domain. Unlike with a pulse basis, where the unknowns are the sound speed on each pixel, this algorithm solves for the limited number of basis function coefficients. Once found, the speed is evaluated at any location in space. The images shown in Figure 4 are cross-sections of the resulting basis function reconstructions at the centroid of each element on a tetrahedral mesh. For the reconstruction of Phantom 1, a fourth-order polynomial basis was used. For Phantom 2, the polynomial order was increased to 5 to support the reconstruction of finer features of the fibroglandular phantom. The images shown were thresholded between 1430 and 1570 m/s. For reference, a schematic showing the true layout of each phantom in the same cross-section is provided in Figure 5.

As seen in the figures, the US reconstructions provide a fair approximation of the physical structure of the tissue regions. However, the reconstructed sound speeds do not agree exactly with the measured values. The maximum reconstructed speeds for Phantoms 1 and 2 are 1542 and 1580 m/s, respectively, whereas the maximum measured speeds of the phantoms are 1587 and 1595 m/s, respectively. Additionally, the reconstructed speeds were observed to increase with the order of polynomial basis functions used. Higher order bases were observed to produce artefacts at the boundaries of the imaging domain [16]. Thus, the quantitative USI technique adopted here, on its own, would not be sufficient to distinguish the tumour from healthy tissue in a real scenario. This is partly due to the minimal number of transducers in the current US data acquisition system.

### 3.2. Sound Speed to Permittivity Mappings

**Figure 6:**
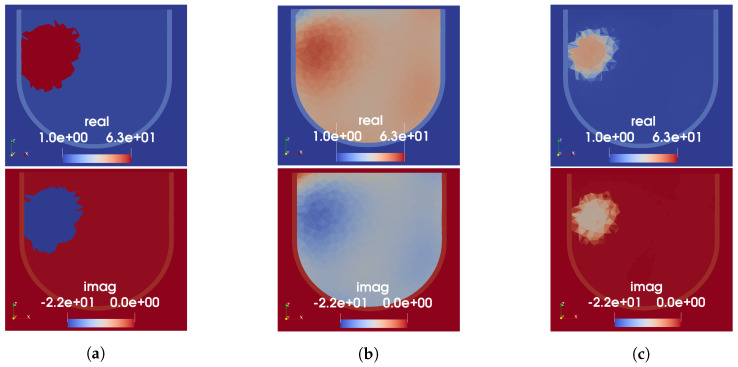
US-derived permittivity maps for Phantom 1. Each column shows real (top) and imaginary (bottom) parts of relative permittivity. (**a**) Segmented mapping. (**b**) Linear mapping. (**c**) Tissue-range mapping.

The three techniques described in Section 2.5 were used to produce initial guess permittivity maps from the reconstructed sound speed images shown in Figure 4. A cross-section of the resulting 3D permittivity maps for Phantom 1 is shown in Figure 6. Note that the skin is not visible in the US reconstructions as it is physically present for both incident and total field measurements. To include the skin in the MW model, a 3 mm thick homogeneous skin region having the same permittivity as the measured skin phantom is added to the prior information after the speed-to-permittivity mapping. In all three cases, a higher permittivity region is present in the prior information in the approximate location of the tumour; however, its permittivity value varies depending on the type of mapping used. The segmented mapping assigns the highest permittivity value to the tumour and also overestimates its size. This enlargement can be attributed to the blurring around the reconstructed tumour in the US image, caused by using polynomial basis functions to approximate a step discontinuity between the fat and tumour regions. The tissue-range mapping produces the smallest sized and lowest-valued estimate of permittivity in the tumour region. Notably, using linear mapping, the fat region is assigned an erroneously high permittivity of approximately 40-j12. This is not the case with the segmented or tissue-range mappings.

**Figure 7:**
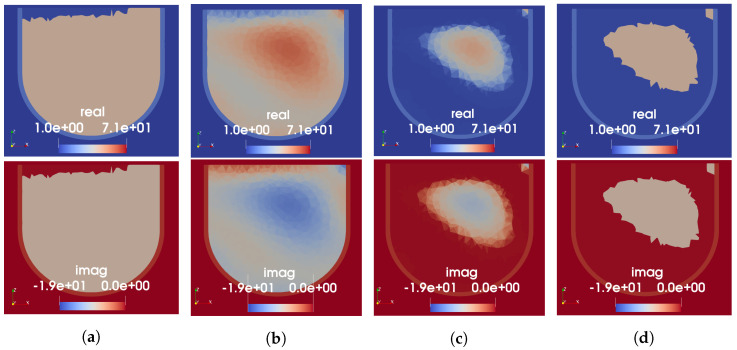
US-derived permittivity maps for Phantom 2. Each column shows the real (top) and imaginary (bottom) parts of relative permittivity. (**a**) Segmented mapping. (**b**) Linear mapping. (**c**) Tissue-range mapping. (**d**) Segmented mapping with low-speed values clipped.

For Phantom 2, because the USI on its own is not expected to recover the tumour due to the lack of sound speed contrast with the fibroglandular region, the number of regions for the segmented mapping is kept at two. The cluster with lower speed is assigned the measured permittivity value of the fat-mimicking material and the higher speed cluster assigned that of the fibroglandular phantom material. The resulting permittivity prior for all three mapping techniques are shown in Figure 7. As seen in Figure 7a, the low-speed artefact at the top of the US reconstruction severely impacts the segmented mapping; no approximation to the fibroglandular region is included in the prior information using this technique. Both the linear and tissue-range mappings preserve some of the fibroglandular structure produced by the US reconstruction. Similar to the results for Phantom 1, only the linear mapping assigns unrealistically high permittivity values to the fat region. The extreme-speed artefacts at the top of the US image were (observed as) present in many US sound speed reconstructions, becoming more apparent as the order of the whole-domain basis function is increased. We presume the artefact to be due to the physical construction of the US fixture: when installed, there is a 1 cm gap between the top of the breast phantom (closest to the chest wall on a real patient) and the nearest US transducers. Thus, the recovered basis function solution is used to estimate the speed of sound in a region where US rays do not intersect the phantom. This is a known source of error in our US reconstructions. In Figure 7d, we show the result of the segmented mapping when reconstructed sound speed values lower than 1450 m/s—slightly lower than the measured minimum speed of the phantom—are clipped prior to the mapping. In this case, the fibroglandular structure becomes visible in the prior information. The linear and tissue-range mappings appear to be more robust to the presence of artefacts in the US reconstructions, though they do propagate some amount of error as seen at the top of the cross-sections shown in Figure 7b,c.

### 3.3. Microwave Reconstruction Results

A selection of the complex permittivity maps shown in the previous section was incorporated into the FEM-CSI algorithm as an initial guess. In this section, we compare the quality of the resulting images when different types of prior information are used. As a comparison to the dual-mode results, we also provide the MWI results obtained using the FEM-CSI algorithm but without US-derived prior information. For this case, the MW measurement data obtained with just the skin and fat regions of the phantom are utilized as the incident measured field, $U^{inc}$, and that phantom is used as the inhomogeneous numerical background in FEM-CSI. Note that this results in a considerable advantage for the reconstruction algorithm; as was investigated in [21], the closer the background phantom is to the OI, the better the ability to reconstruct the difference between the two. While it is of course not possible to image a real breast with and without a tumour (with the possible exception of the tumour monitoring application), a similar measurement could be obtained by filling the future breast-support cup with a substance that mimics the bulk-average complex permittivity of the breast being imaged.

#### 3.3.1. Phantom 1

**Figure 8:**
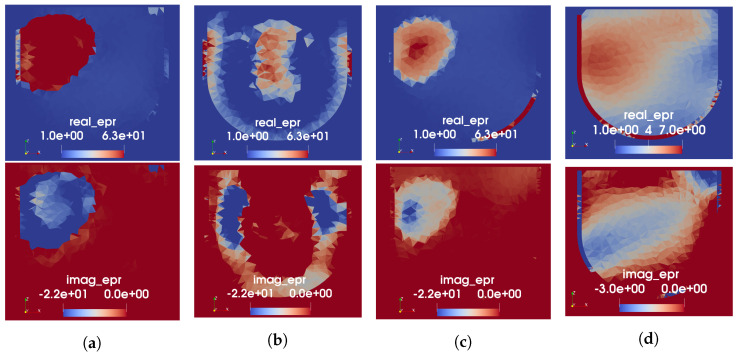
FEM-CSI reconstructions at 1.0 GHz for Phantom 1. (**a**) Initial guess from segmented mapping. (**b**) Initial guess from linear mapping. (**c**) Initial guess from tissue-range mapping. (**d**) Perfect prior of skin and fat only (no USI).

The FEM-CSI reconstruction results for Phantom 1 obtained using four different variations of prior information are shown in Figure 8. Results from the three different speed-to-permittivity mappings used as the initial guess for CSI are shown in subfigures a–c, while the MW-only inversion that had perfect prior of the skin and fat is shown in Figure 8d. Each subfigure shows the same axial slice of the 3D complex-valued permittivity image, with a real part in the top row and the imaginary on the bottom. Note that the colour bars for the MW-only images are not the same as those shown for the dual-mode images; the reconstructed permittivity in the tumour region is considerably lower in the MW-only case, approximately 6-j2. For all cases, the imaging domain was restricted to within the breast, i.e., elements outside the provided skin region were not allowed to vary. The allowable reconstructed values for complex permittivity were constrained to $1\leq \epsilon^{\prime }\leq 70$ and $0\leq \epsilon^{\prime \prime }\leq 30$.

Evaluation of the resulting images has, thus far, been limited to qualitative analysis. From the images in Figure 8, it appears that the segmented and tissue-range mappings yield significantly more accurate images than the linear mapping, with which no tumour is distinguishable. While the MW-only reconstruction produces a region of higher permittivity in the approximate location of the tumour, its shape is blurred, and permittivity is quite low. Furthermore, the shape and location of the reconstructed tumour are more accurate in the real part of permittivity than it is in the imaginary. This discrepancy between the real and imaginary parts appears to be mitigated when the US-derived prior information from the segmented or tissue-range mappings is used as an initial guess. The poor results obtained using the prior information from the linear mapping may be due to the incorrectly high permittivity assigned to the fat region by this mapping, as noted previously. This explanation agrees with the observation published in [11], where lower-valued permittivity prior resulted in increased sensitivity for tumour detection. In comparing the results obtained using the segmented versus tissue-range mappings, the reconstructed values of the tumour permittivity differ. In the permittivity map generated using the segmented mapping, the real part of the tumour had a maximum value of 63. The permittivity of the tumour after the MW reconstruction goes up to 70, the upper bound imposed on $\epsilon^{\prime }$. In the permittivity map generated with the tissue-range mapping, the real part of the tumour had a maximum value of 49. As seen here, FEM-CSI is able to increase this value to 60. This is a considerable improvement from the reconstructed tumour permittivity when US-derived prior information is not used.

Regardless of the form of the prior information, the skin seemingly disappears in some spots and has high permittivity in other spots. From a tumour-detection point of view, this is undesirable as it would lead to false positives within the skin region. Thus, for the upcoming reconstruction results for Phantom 2, the skin region was excluded from the imaging domain.

#### 3.3.2. Phantom 2

**Figure 9:**
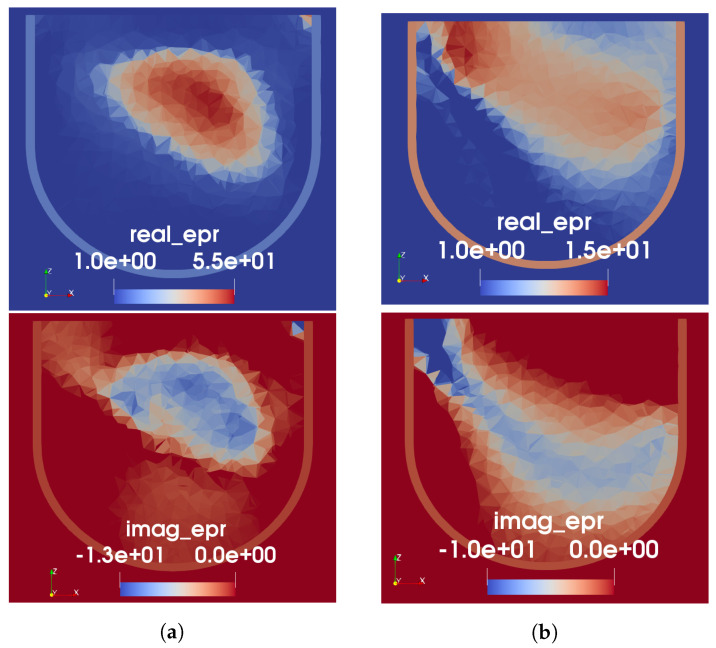
FEM-CSI reconstructions for Phantom 2. (**a**) Initial guess from tissue-range mapping. (**b**) Perfect prior of skin and fat only (no USI).

Based on the results seen for Phantom 1 and the difficulty with the segmented mapping for Phantom 2, the tissue-range mapping was selected as the technique for producing prior information for Phantom 2. Thus, the permittivity map depicted in Figure 7c was used as the initial guess for the FEM-CSI algorithm. This time, a simultaneous frequency implementation of the algorithm was used with data collected at 1.0 and 1.3 GHz. As explained in the previous section, the permittivities of elements within the skin region were not allowed to vary. A cross-section of the resulting 3D image is shown in Figure 9a. As a comparison, we show the reconstructed image when US-derived prior information is not used in Figure 9b. As with Phantom 1, perfect prior information of the geometry and permittivity of the skin and fat regions only were provided in this case. No prior knowledge about the fibroglandular or tumour was assumed.

As seen from the images in Figure 9, the US-derived prior information clearly results in improved MW reconstruction of the interior structure of the breast phantom, compared to when prior information of only the skin and fat is provided. For the MW-only inversion, while there are regions of higher permittivity in roughly the expected location of the fibroglandular tissue, the peak value in the reconstruction is low: 19-j15. Additionally, as seen with Phantom 1, the reconstructed real part of permittivity is more accurate than the imaginary. When the US-derived prior information is used as an initial guess, it can be observed that the MWI produces some structure within the given fibroglandular region. The peak value in the reconstruction, in this case, is 55-j13; this is still lower than the measured value; however, we have found that reconstructions of high-contrast targets typically undershoot the true permittivity.

## 4. Discussion

The results presented in this paper demonstrate the feasibility of a novel experimental integrated 3D dual-mode microwave-ultrasound breast imaging system. A complete workflow of taking low-resolution US speed reconstructions, converting them to complex-valued permittivity, and incorporating them as prior information for MWI has been described. Preliminary testing of this workflow was performed using two simplified breast phantoms to validate the soundness of the imaging procedure. Three different methods for generating prior information from the US reconstruction for use in the MW inversion algorithm have been investigated. Though there is room for modifications to be made to the sound speed to permittivity mappings, we conclude that the tissue-range mapping is highly beneficial due to its robustness to sound speed artefacts and the ability to map all tested tissue types to realistic permittivity values.

Although not presented herein, the utility of the resulting images to enhance tumour detection was investigated in [16,23] using a simple thresholding technique. Two methods of incorporating the US-derived prior information were studied: the initial guess technique used in this work, as well as incorporating the prior as an inhomogeneous background. More research is required into tumour detection algorithms, but the methodology developed in this work enables such research and represents a significant milestone in the field of dual-mode MW-US breast imaging.

Our future work includes the development of more realistic dual-mode tissue-mimicking breast phantoms so that the imaging procedure can be more thoroughly evaluated. The value of constructing higher resolution US images is an open question, although such reconstructions may require a more elaborate US data acquisition fixture. Alternatively, using the same low-resolution data, the resolution of the US images could be improved by reconstructing additional US parameters, such as attenuation, or the use of other, qualitative reconstruction techniques, such as DAS [13,35]. The creation of the complex-valued permittivity maps from the US images can also be improved using deep-learning techniques [36]. In addition to the two techniques for incorporating prior information for MWI referenced above, other methodologies, such as regularization using spatial prior [8,37], or joint inversion [19,38,39], although only developed using synthetic data, are promising.

Any future clinical investigation would require some modifications to our current system. Perhaps the largest hurdle to overcome is in the design and fabrication of a suitable breast-support cup, or cups, that could be used during both MW and US data collection. To accommodate the wide possible range of patients, support cups of various shapes and sizes could be manufactured and even custom-fitted to a patient’s breast. Additionally, for both modalities, we will require a calibration object and background incident field that are good approximations of the patient’s breast. As it is unlikely that we will have access to measured data for the same breast with and without a tumour, the use of a breast phantom with physical properties that approximate the bulk-average properties of a patient’s breast could be used. This phantom may consist of the breast-support cup filled with some material that approximates the bulk MW and US properties. This would require a study of how the mismatch between the phantom properties and the true properties of the breast affects the reconstruction quality.

## Figures and Tables

**Table 1:** Ingredients and measured physical properties of dual-mode breast phantom materials. Target values are in italics.

	Skin	Fat	Fibroglandular	Tumour
**Ingredients**	graphite,	canola oil	50 mL water	100 mL water
	urethane,		50 mL 1-propanol	1/8 tsp table salt
	as given in [24]		7 g gelatin	7 g gelatin
			3 g agar	3 g agar
			25 mL glycerin	12.5 mL glycerin
**Sound Speed**	not measured	1460	1595	1587
m/s, at 1.4 MHz		*(1470)*	*(1515)*	*(1555)*
**Relative Permittivity**	11-j1.3	2.9-j0.23	41-j8.4	63-j22, Phantom 1
at 1 GHz	*(35-j23)*	*(13-j10)*	*(33-j21)*	71-j19, Phantom 2
				*(53-j19)*

**Table 2:** Property ranges used in tissue-range mapping technique for Phantom 1.

Tissue Type	Speed of Sound	$\epsilon_{\mathrm{real}}$	$\epsilon_{\mathrm{imag}}$
Fat	1430–1480 m/s	2.5–3.5	0.15–0.25
Transitional	1481–1499	3.51–40.0	0.26–9.49
Fibro	1500–1559	40.01–52.5	9.50–12.50
Tumour	1560–1600	52.51–66.98	12.51–25.17

## Data Availability

All images were generated using algorithms, the details of which have been publicly disclosed. The computer implementation of these algorithms is not available for the public at large. Synthetic data computed with these algorithms are available upon request. Experimental data collected using the experimental systems described are also available upon request.

## Abbreviations

The following abbreviations are used in this manuscript:

AIC	Akaike information criterion
AWG	arbitrary waveform generator
CCT	computer-controlled transceiver
CSI	contrast source inversion
FEM	finite element method
MER	modified energy ratio
MRI	magnetic resonance imaging
MWI	microwave imaging
MWR	microwave radar
OI	object of interest
PC	personal computer
TOF	time-of-flight
USI	ultrasound imaging
VNA	vector network analyzer

## References

[1] Bolomey J.C. (2018). Crossed viewpoints on microwave-based imaging for medical diagnosis: From genesis to earliest clinical outcomes. The World of Applied Electromagnetics.

[2] Moloney B.M., O’Loughlin D., Abd Elwahab S., Kerin M.J. (2020). Breast cancer detection—A synopsis of conventional modalities and the potential role of microwave imaging. Diagnostics.

[3] Mojabi P., LoVetri J. (2019). Experimental evaluation of composite tissue-type ultrasound and microwave imaging. IEEE J. Multiscale Multiphys. Comput. Tech..

[4] O’Loughlin D., O’Halloran M., Moloney B.M., Glavin M., Jones E., Elahi M.A. (2018). Microwave breast imaging: Clinical advances and remaining challenges. IEEE Trans. Biomed. Eng..

[5] Boverman G., Davis C.E., Geimer S.D., Meaney P.M. (2017). Image registration for microwave tomography of the breast using priors from nonsimultaneous previous magnetic resonance images. IEEE J. Electromagn. Microwaves Med. Biol..

[6] Kurrant D., Fear E. (2012). Regional estimation of the dielectric properties of inhomogeneous objects using near-field reflection data. Inverse Probl..

[7] Omer M., Mojabi P., Kurrant D., LoVetri J., Fear E. (2018). Proof-of-concept of the incorporation of ultrasound-derived structural information into microwave radar imaging. IEEE J. Multiscale Multiphys. Comput. Tech..

[8] Golnabi A.H., Meaney P.M., Geimer S.D., Paulsen K.D. (2019). 3D microwave tomography using the soft prior regularization technique: Evaluation in anatomically realistic MRI-derived numerical breast phantoms. IEEE Trans. Biomed. Eng..

[9] Jiang H., Li C., Pearlstone D., Fajardo L.L. (2005). Ultrasound-guided microwave imaging of breast cancer: Tissue phantom and pilot clinical experiments. Med. Phys..

[10] Bayat N., Mojabi P., Lovetri J., Mojabi P. On Microwave Breast Imaging with Ultrasound Spatial Priors. Proceedings of the 2020 XXXIIIrd General Assembly and Scientific Symposium of the International Union of Radio Science.

[11] Abdollahi N., Kurrant D., Mojabi P., Omer M., Fear E., LoVetri J. (2019). Incorporation of Ultrasonic Prior Information for Improving Quantitative Microwave Imaging of Breast. IEEE J. Multiscale Multiphys. Comput. Tech..

[12] Mojabi P., Abdollahi N., Omer M., Kurrant D., Jeffrey I., Fear E., LoVetri J. Tissue-Type Imaging for Ultrasound-Prior Microwave Inversion. Proceedings of the 2018 18th International Symposium on Antenna Technology and Applied Electromagnetics (ANTEM).

[13] Duric N., Littrup P., Poulo L., Babkin A., Pevzner R., Holsapple E., Rama O., Glide C. (2007). Detection of breast cancer with ultrasound tomography: First results with the Computed Ultrasound Risk Evaluation (CURE) prototype. Med. Phys..

[14] Duric N., Littrup P., Kuzmiak C. (2018). Breast ultrasound tomography. Breast Imaging.

[15] Duric N., Sak M., Fan S., Pfeiffer R.M., Littrup P.J., Simon M.S., Gorski D.H., Ali H., Purrington K.S., Brem R.F. (2020). Using whole breast ultrasound tomography to improve breast cancer risk assessment: A novel risk factor based on the quantitative tissue property of sound speed. J. Clin. Med..

[16] Fogel H. (2022). Development of a Dual-Mode Microwave-Ultrasound Breast Imaging System. Master’s Thesis.

[17] Khoshdel V., Ashraf A., LoVetri J. (2019). Enhancement of multimodal microwave-ultrasound breast imaging using a deep-learning technique. Sensors.

[18] Qin Y., Rodet T., Lambert M., Lesselier D. (2020). Microwave breast imaging with prior ultrasound information. IEEE Open J. Antennas Propag..

[19] Qin Y., Rodet T., Lambert M., Lesselier D. (2021). Joint inversion of electromagnetic and acoustic data with edge-preserving regularization for breast imaging. IEEE Trans. Comput. Imaging.

[20] Hughson M. (2021). Quantitative Transmission Tomography for Non-Destructive Imaging of Stored Grain and Biological Tissue. Master’s Thesis.

[21] Asefi M., Baran A., LoVetri J. (2019). An experimental phantom study for air-based quasi-resonant microwave breast imaging. IEEE Trans. Microw. Theory Tech..

[22] Nemez K., Baran A., Asefi M., LoVetri J. (2017). Modeling error and calibration techniques for a faceted metallic chamber for magnetic field microwave imaging. IEEE Trans. Microw. Theory Tech..

[23] Fogel H.C., Hughson M., Asefi M., Jeffrey I., LoVetri J. An Integrated Microwave-Ultrasound Breast Imaging System: Initial Phantom Results. Proceedings of the 2022 16th European Conference on Antennas and Propagation (EuCAP).

[24] Garrett J.D. (2014). Average Dielectric Property Analysis of Non-Uniform Structures: Tissue Phantom Development, Ultra-Wideband Transmission Measurements, and Signal Processing Techniques. Master’s Thesis.

[25] Medina-Valdés L., Pérez-Liva M., Camacho J., Udías J., Herraiz J., González-Salido N. (2015). Multi-modal ultrasound imaging for breast cancer detection. Phys. Procedia.

[26] Kaye C., Jeffrey I., LoVetri J. (2019). Improvement of multi-frequency microwave breast imaging through frequency cycling and tissue-dependent mapping. IEEE Trans. Antennas Propag..

[27] Wong J., Han L., Bancroft J., Stewart R. (2009). Automatic time-picking of first arrivals on noisy microseismic data. CSEG. 0 0.2 0.4 0.6 0.8.

[28] Liu K.Y., Fear E.C., Potter M.E. (2015). Antenna aperture localization for arrival time correction using first-break. Prog. Electromagn. Res..

[29] St-Onge A. (2011). Akaike information criterion applied to detecting first arrival times on microseismic data. SEG Technical Program Expanded Abstracts 2011.

[30] Zakaria A., Gilmore C., LoVetri J. (2010). Finite-element contrast source inversion method for microwave imaging. Inverse Probl..

[31] Amer Zakaria I.J., LoVetri J., Zakaria A. (2013). Full-vectorial parallel finite-element contrast source inversion method. Prog. Electromagn. Res..

[32] Kurrant D., Baran A., LoVetri J., Fear E. (2017). Integrating prior information into microwave tomography Part 1: Impact of detail on image quality. Med. Phys..

[33] Kurrant D., Fear E., Baran A., LoVetri J. (2017). Integrating prior information into microwave tomography part 2: Impact of errors in prior information on microwave tomography image quality. Med. Phys..

[34] Ostadrahimi M., Mojabi P., Gilmore C., Zakaria A., Noghanian S., Pistorius S., LoVetri J. (2011). Analysis of incident field modeling and incident/scattered field calibration techniques in microwave tomography. IEEE Antennas Wirel. Propag. Lett..

[35] Pérez-Liva M., Herraiz J., Udías J., Miller E., Cox B., Treeby B. (2017). Time domain reconstruction of sound speed and attenuation in ultrasound computed tomography using full wave inversion. J. Acoust. Soc. Am..

[36] Mojabi P., Hughson M., Khoshdel V., Jeffrey I., LoVetri J. (2021). CNN for compressibility to permittivity mapping for combined ultrasound-microwave breast imaging. IEEE J. Multiscale Multiphys. Comput. Tech..

[37] Bayat N., Mojabi P. (2021). A Multiplicative Regularizer Augmented with Spatial Priors for Microwave Imaging. IEEE Trans. Antennas Propag..

[38] Song X., Li M., Yang F., Xu S., Abubakar A. (2019). Study on joint inversion algorithm of acoustic and electromagnetic data in biomedical imaging. IEEE J. Multiscale Multiphys. Comput. Tech..

[39] Qin Y., Ran P., Rodet T., Lesselier D. (2022). Breast imaging by convolutional neural networks from joint microwave and ultrasonic data. IEEE Trans. Antennas Propag..

